# An eight-year epidemiologic study based on baculovirus-expressed type-specific spike proteins for the differentiation of type I and II feline coronavirus infections

**DOI:** 10.1186/s12917-014-0186-7

**Published:** 2014-08-15

**Authors:** Ying-Ting Wang, Ling-Ling Chueh, Cho-Hua Wan

**Affiliations:** 1 Graduate Institute of Veterinary Medicine, School of Veterinary Medicine, National Taiwan University, Taipei 10617, Taiwan; 2 Graduate Institute of Molecular and Comparative Pathobiology, School of Veterinary Medicine, National Taiwan University, Taipei 10617, Taiwan

**Keywords:** Seroprevalence, Feline coronavirus, Baculovirus, Recombinant protein

## Abstract

**Background:**

Feline infectious peritonitis (FIP) is a fatal disease caused by feline coronavirus (FCoV). FCoVs are divided into two serotypes with markedly different infection rates among cat populations around the world. A baculovirus-expressed type-specific domain of the spike proteins of FCoV was used to survey the infection of the two viruses over the past eight years in Taiwan.

**Results:**

An immunofluorescence assay based on cells infected with the recombinant viruses that was capable of distinguishing between the two types of viral infection was established. A total of 833 cases from a teaching hospital was surveyed for prevalence of different FCoV infections. Infection of the type I FCoV was dominant, with a seropositive rate of 70.4%, whereas 3.5% of cats were infected with the type II FCoV. In most cases, results derived from serotyping and genotyping were highly agreeable. However, 16.7% (4/24) FIP cats and 9.8% (6/61) clinically healthy cats were found to possess antibodies against both viruses. Moreover, most of the cats (84.6%, 22/26) infected with a genotypic untypable virus bearing a type I FCoV antibody.

**Conclusion:**

A relatively simple serotyping method to distinguish between two types of FCoV infection was developed. Based on this method, two types of FCoV infection in Taiwan was first carried out. Type I FCoV was found to be predominant compared with type II virus. Results derived from serotyping and genotyping support our current understanding of evolution of disease-related FCoV and transmission of FIP.

## Background

Feline coronavirus (FCoV), a common pathogen in cats, is an enveloped, positive-sense, single-stranded RNA virus. Together with canine coronavirus (CCoV), transmissible gastroenteritis virus (TGEV), porcine respiratory coronavirus and human coronavirus 229E (HCoV 229E), FCoV is classified into the genus *Alphacoronavirus*[1]. Two pathotypes of FCoV have been well demonstrated, i.e., feline enteric coronavirus (FECV) and feline infectious peritonitis (FIP) virus (FIPV). The former causes mild enteric infections, and the latter causes a fatal immune-mediated disease known as FIP [2].

Infection with coronavirus is determined by the interaction between the receptor binding domain of its spike (S) protein and corresponding receptors on target cells. S protein is the main determinant of cell tropism and of the induction of neutralizing antibodies [3]. FCoVs can be divided into two serotypes, types I and II, which differ in the neutralizing antibody reaction and have distinct S protein sequences [4]. These two types of FCoV differ in growth characteristics *in vitro*. Type II FCoV is antigenically related to CCoV and TGEV [5]. The receptor usage of the two types of FCoV is different [6]. Feline aminopeptidase N was identified as a receptor for type II FCoV, but not for type I FCoV [7]. The main receptor for type I FCoV infection remains unclear. A feline dendritic cell-specific intercellular adhesion molecule-grabbing nonintegrin serves as a coreceptor for both type I and II FCoV [8]. Both types of FCoV can infect domestic and wild *Felidae* and cause FIP [2]. Seroprevalence studies for the detection of the two types of FCoV infection have been performed using various methods, and type I FCoV was found to be predominant in the field, with a seropositive rate of 83-98%, whereas the type II virus accounted for only less than 10% of infections [9]-[11].

In this study, a type-specific partial S protein-based immunofluorescence assay (IFA) was established to distinguish between the two serotypes of FCoV. The seroprevalence of FCoV in Taiwan was determined, and the correlation between the genotypes and serotypes of FCoV infection was assessed.

## Methods

### Viruses and cells

For the cultivation, purification and titration of recombinant baculovirus (r-virus), *Spodoptera frugiperda*-9 (*Sf*-9) cells were used in this study. The *Sf*-9 cells were cultured in suspension at 27°C at densities ranging from 0.5-2 × 10^6^ cells/ml in HyQ SFX-Insect Media (HyClone, Logan, UT, USA) containing 5% (v/v) fetal bovine serum (FBS) (PAA Laboratories GmbH, Pasching, Austria) and 10 μg/ml gentamicin.

*Felis catus* whole fetus-4 (Fcwf-4) cells were used for the propagation of the type II FCoV strain NTU156 [12] and maintained in Dulbecco’s modified Eagle’s medium (Gibco, Grand Island, USA) supplemented with 10% FBS, 100 IU/ml penicillin and 100 μg/ml streptomycin in 5% CO_2_ at 37°C.

### Detection and genotyping of FCoV

Several samples were collected from the cats enrolled in this study, including whole blood, plasma, swab samples (rectal, nasal, oral and conjunctival swabs), body effusions and internal organ samples, and were screened for FCoV by reverse transcription-nested polymerase chain reaction (RT-nPCR) [13]. FCoV-positive samples were subsequently subjected to genotyping of the virus according to the procedures reported by Addie et al. [14].

### Clinical samples

To further characterize the correlation between the serotype of the infection and FIP, plasma samples from 43 pathologically confirmed, naturally occurring FIP cases and 30 suspected FIP cases, which were FCoV RT-nPCR positive in effusions with/without an IFA signal present in macrophages [15], were further tested. Moreover, to evaluate the seroprevalence of different types of FCoV infection in Taiwan, plasma samples from 760 clinically healthy cats were collected around the island of Taiwan from 1996–2013. All samples were stored at −20°C until analysis. An ethical approval was not required as this study was performed retrospectively and samples of diseased animals were routinely submitted to the veterinary diagnostic laboratory.

### Detection of anti-FCoV antibody

For the screening of anti-FCoV antibody-positive samples, a type II FCoV-based IFA was used in the present study. Fcwf-4 cells seeded in a 96-well plate were infected with the type II FCoV strain NTU156 at a multiplicity of infection (MOI) of 0.01 and incubated for 18 hours. Once each well contained 30% infected cells and 70% uninfected cells as an internal negative control, the infected cells were fixed with cold 50/50 acetone/methanol (v/v) for ten minutes. A plasma sample diluted 1:40 with PBS was transferred to the well and allowed to incubate for 60 minutes at room temperature. Subsequently, the cells were washed 5 times with PBS, and fluorescein isothiocyanate (FITC)-conjugated goat anti-cat IgG (1:1000 dilution) was added to each well for a 60-minute incubation at room temperature. When syncytial cells, which are a typical cytopathic effect (CPE) of FCoV infection, showed fluorescent signals that were interpreted as positive, the cat was considered to be infected with FCoV. Positive samples were further serotyped.

### Amplification, cloning and expression of type-specific *S* genes

Because S protein is the key determinant of the discrimination of serotypes, a region specific to the two types of FCoV was chosen for analysis. The type I FCoV strain NTU2 (GenBank: DQ160294) and the type II FCoV strain NTU156 (GenBank: GQ152141) were employed in this study. Two pairs of primers based on the RBD region of the *S* gene, with the restriction site EcoR1, Kpn1 or BamH1, were designed (FCoV-I pRBD F: 5’-*gaattc*atgttttactctgctagtatgctt-3’ and FCoV-I pRBD R: 5’-*ggtacc*ttaacccagctgtgcctttg-3’; FCoV-II pRBD F: 5’-*ggatcc*atgcctgtagcctcgagtgacg-3’ and FCoV-II pRBD R: 5’-*ggtacc*ttagtgaggaccactattatcagac-3’) and used to amplify the targeted region of the *S* gene. This region was 510 and 513 nucleotides in length for type I and II FCoV, respectively. The amplified products were cloned into the pFastBac™ HT vector using the Bac-to-Bac Baculovirus Expression System (Invitrogen srl, Milan, Italy) following the manufacturer’s instructions. The FCoV-I pRBD-bacmid and FCoV-II pRBD-bacmid were used to transfect *Sf-9* cells to obtain r-viruses (rFCoV-I and rFCoV-II) containing an N-terminal 6× histidine tag.

### Confirmation of recombinant proteins

*Sf*-9 cells were infected with rFCoV-I or rFCoV-II at different MOI values, and cell pellets from different post-infection times were lysed for Western blotting analysis. The expressed recombinant proteins were resolved by SDS-PAGE and transferred to PVDF membranes. The membranes were blocked using 5% (w/v) non-fat dry milk for two hours at room temperature, followed by incubation with an anti-histidine mouse monoclonal antibody (mAb) (Invitrogen srl, Milan, Italy) at room temperature. After washes in PBS-Tween 20, each membrane was incubated with horseradish peroxidase (HRP)-conjugated goat anti-mouse IgG antibody (Jackson ImmunoResearch Laboratories, PA, USA). The membranes were developed with 3,3’,5,5’-tetramethylbenzidine (TMB) substrate (Kirkegaard & Perry Laboratories, MD, USA).

### Serotyping

To differentiate the serotypes of infection, a novel type-specific IFA using rFCoV-I- or rFCoV-II -infected *Sf-9* cells was established. *Sf-9* cells seeded in a 96-well plate were infected with either rFCoV-I or rFCoV-II at an MOI of 0.5. After 72 hours of incubation, the infected cells were fixed with cold acetone/methanol. Two-fold-diluted plasma samples (from 1:50 to 1:1600) from cats with FIP cats were transferred into the wells (rFCoV-I- or and rFCoV-II -infected *Sf-9* cells or mock infection) and incubated for 60 minutes at room temperature. After washing 5 times with PBS, 1000-fold-diluted FITC-conjugated goat anti-cat IgG was added to each well for 60 minutes at room temperature. FCoV-positive sera collected from cats without FIP were diluted 1:100 and applied to the IFA system as above for serotyping analysis. Two polyclonal antisera (VMRD, Pullman, WA, USA) against type I and II FCoV were used as referenced positive-control sera. Two sera from specific pathogen-free (SPF) cats were used as negative-control sera. The serotype of each FCoV infection was identified based on rFCoV-I- or rFCoV-II-infected *Sf-9* cells (rRBD-1 and rRBD-II) showing a fluorescent signal after IFA staining with 200-fold-diluted or higher plasma samples from cats with FIP and 100-fold-diluted samples from healthy cats.

### Neutralization test (NT)

To further compare the assay established in this study to a gold standard method, plasma samples from FIP confirmed animals were examined by both NT test and the type-specific IFA. Heat inactivated plasma samples were diluted 10 and 100 fold followed by a two fold serial dilution up to 1600 fold. Subsequently, diluted sample were mixed with an equal volume of a type II FCoV strain NTU156 containing 100 PFU/50 μl and incubated at 37°C for 1 h. Each mixture was then inoculated onto Fcwf-4 cells monolayer in a 96 well microplate at 37°C for 3 days. The reciprocal of the highest dilution of antibody that completely inhibited viral CPE formation was expressed as the virus-neutralizing titer.

## Results

### Prevalence of FCoV in Taiwan

All of the samples from the 43 confirmed FIP cases and 30 suspected FIP cases were FCoV seropositive. In addition to the cats with FIP, a total of 760 clinically healthy cats collected from 26 veterinary hospitals in 6 cities in Taiwan over 8 years were enrolled in this study, 28.2% (214/760) of which were positive for FCoV. The overall prevalence of FCoV in Taiwan was 34.5% (287/833) (Table 1).

**Table 1:** Prevalence of FCoV serotypes I and II in cats with different disease statuses in Taiwan

**Status**	**Serotype n (%)**				**Total**
	**I**	**II**	**I/II**	**Untypable**	
FIP	29 (67.4)	3 (7.0)	11 (25.6)	0 (0)	43
FIP-suspected	24 (80.0)	2 (6.7)	3 (10.0)	1 (3.3)	30
Non-FIP	149 (69.7)	5 (2.3)	37 (17.3)	23 (10.7)	214
Total	202 (70.4)	10 (3.5)	51 (17.8)	24 (8.3)	287

### Amplification of *S* region based on RBD of type I and II FCoV for type-specific protein expression

**Figure 1:**
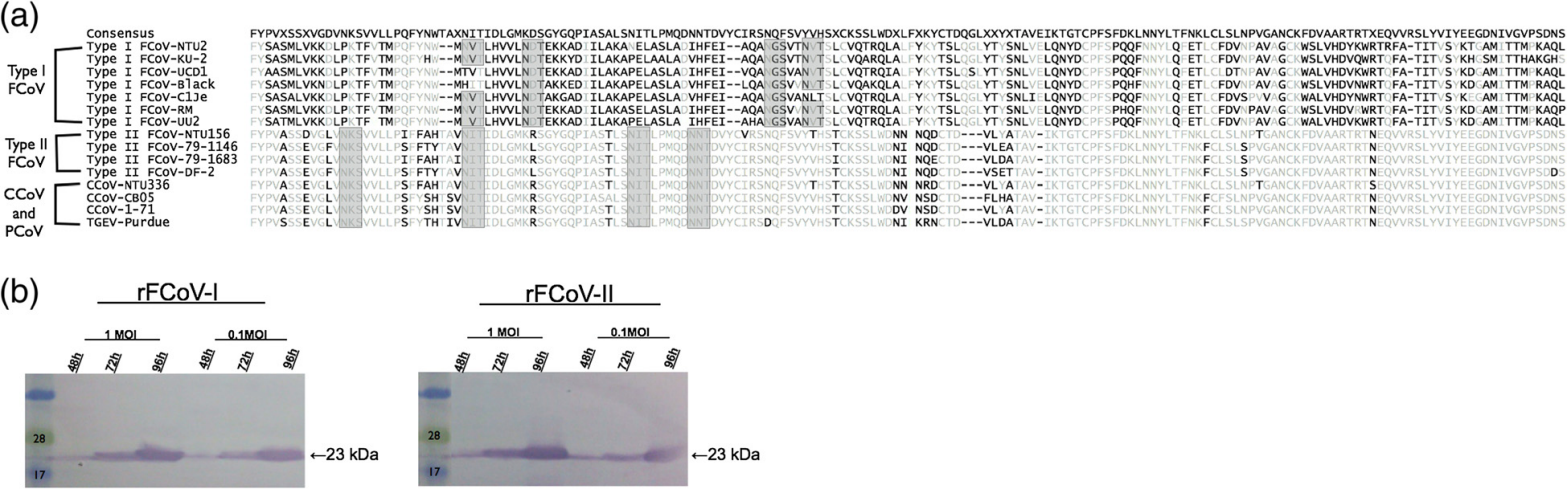
**Amplification and expression of the type-specific S region of type I and II FCoV. (a)** The alignment of the amino acid sequences of the putative RBD of S protein from type II FCoV and type I FCoV with the sequences from prototypic viral strains. The boxed nucleotides represent N-linked glycosylation sites. GenBank accession number: FCoV NTU2 (GenBank: DQ160294), FCoV KU-2 (GenBank: AB086881), FCoV UCD1 (GenBank: AB088222), FCoV Black (GenBank: EU186072), FCoV C1Je (GenBank: DQ848678), FCoV RM (GenBank: FJ938051), FCoV UU-2 (GenBank:FJ938060), FCoV NTU156 (GenBank: GQ152141), FCoV 79–1146 (GenBank: DQ010921), FCoV 79–1683 (GenBank: JN634064), FCoV DF-2 (GenBank: JQ408981), CCoV NTU336 (GenBank: GQ477367), CCoV CB05 (GenBank: DQ112226), CCoV 1–71 (GenBank: JQ404409) and TGEV Purdue (GenBank: DQ811789). **(b)***Sf-9* cells were infected with two r-viruses (rFCoV-I and rFCoV-II) at different MOI values, and the cells were harvested at different times post-infection. The putative RBD of S proteins (23 kDa in size) from type I and type II FCoV were confirmed by Western blotting using an anti-histidine mAb.

To distinguish between the serotypes of FCoV infection, a novel type-specific IFA was established. To express the protein fragment that is antigenically distinct between type I and II FCoV, the type-specific region of the *S* gene, based on the putative RBD of type II FCoV and the corresponding region of type I FCoV, was selected. The expressed regions were located from amino acids no. 523 to no. 692 and no. 508 to no. 678 of the S proteins of type I and II FCoV, respectively (Figure 1a). The partial *S* genes of different types of FCoV were amplified, directionally cloned into the pFastBac™ HT vector and used to generate r-viruses, i.e., rFCoV-I and rFCoV-II. Recombinant proteins with the expected size (approximately 23 kDa for type I and type II FCoV) were identified (Figure 1b).

### Establishment and confirmation of type-specific IFA

**Figure 2:**
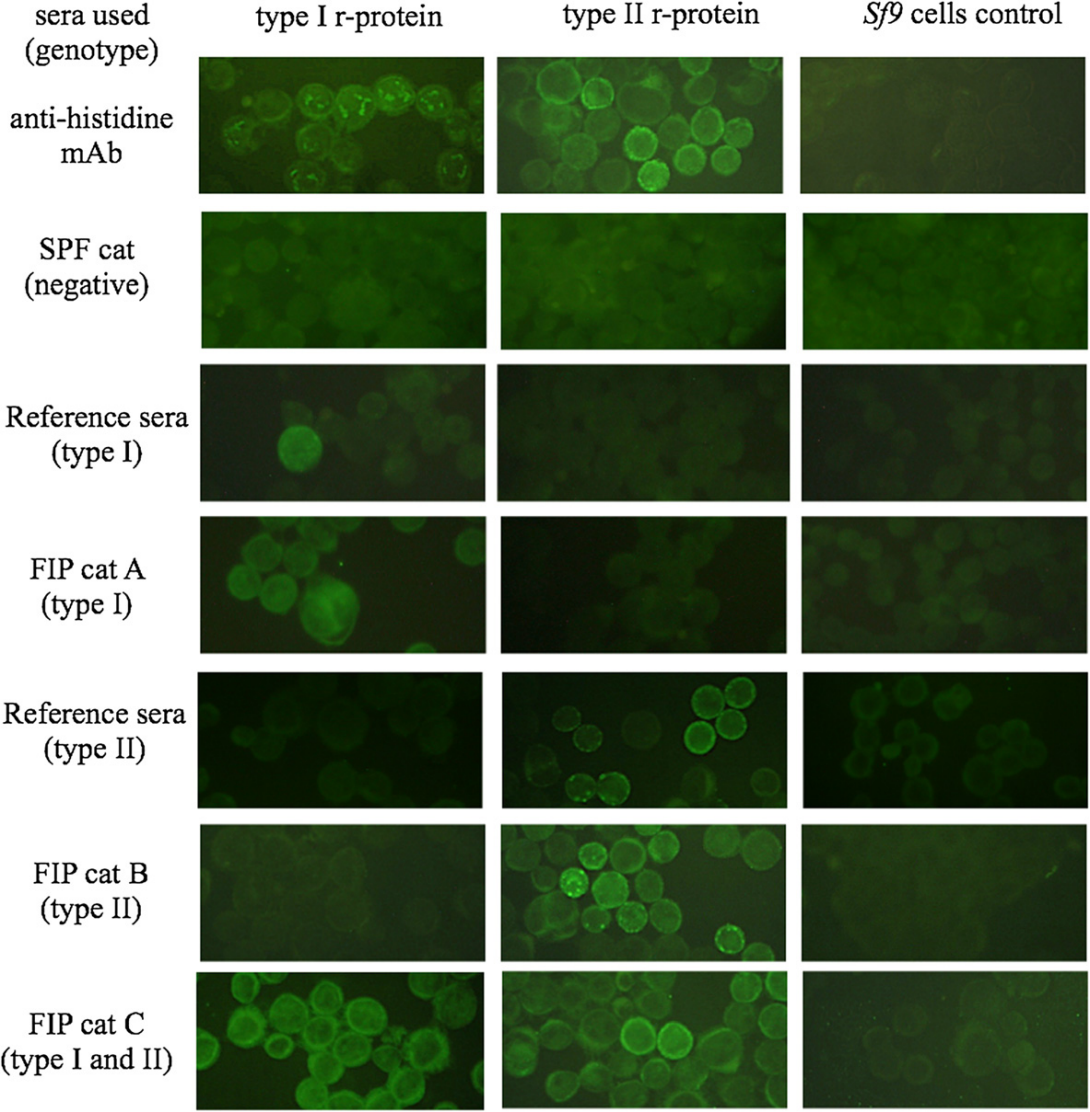
**Establishment and confirmation of the type-specific IFA.** Sera from confirmed type I and/or II FCoV-infected cats with FIP, with type I or II FCoV antisera or SPF cat serum as a reference, were applied to the IFA to characterize its specificity.

The specificity of the type-specific IFA was verified using plasma samples from the confirmed type I or II FCoV-infected cats or from SPF cats. Both recombinant proteins could be detected by the anti-histidine mAb, with a positive signal located in the cytoplasm. In contrast, no fluorescent signal was observed while using sera from the SPF cats (Figure 2). The plasma from type I FCoV-infected cats could specifically react with rRBD-I, but not with rRBD-II, and vice versa. While using the plasma samples from dually FCoV-infected cats with FIP, positive signals could be observed for both types of r-RBD (Figure 2). Based on these data, the specificity of both recombinant proteins was confirmed. To further validate the assay, the serotyping result derived from type-specific IFA was compared to a gold standard, NT. Plasma samples from FIP animals bearing antibody against rRBD-II (≥400×) were found to possess a relatively high NT titer (≥800×) against type II FCoV, indicating a type II FIPV infection (5/5). On the other hand, samples bearing antibody solely against rRBD-I (200 × − 1600×) showed barely detectable NT titer (≤10×) against type II virus, indicating a type I FIPV infection (19/19) (Table 2). The results show that serotypes determined by the type-specific IFA test are highly agreeable with NT test.

**Table 2:** Genotype, serotype and NT titer against FCoV from FIP cats

**Case**	**Genotype**^**a**^		**Serotype (IFA titer)**	**NT titer**^**b**^
	**Typing I (specimen)**	**Typing II (specimen)**		
FIP-1	As		I (400)	≤10
FIP-2	As		I (800)	10
FIP-3	As		I (800)	≤10
FIP-4	As		I (800)	
FIP-5	B, R		I (≥1600)	
FIP-6	Lu		I (400)	
FIP-7	B, R		I (800)	≤10
FIP-8	R		I (800)	
FIP-9	B		I (800)	
FIP-10	R		I (400)	
FIP-11	Pl		I (400)	10
FIP-12	R, Br		I (800)	≤10
FIP-13	R		I (400)	10
FIP-14	Lu		I (400)	
FIP-15	As		I (≥1600)	≤10
FIP-16	As		I (200)	
FIP-17	As		I (400)	10
FIP-18	R		I (400)/II (400)	
FIP-19	As, R		I (400)/II (400)	
FIP-20	As		I (≥1600)/II (400)	
FIP-21		As, B, R	II (≥1600)	
FIP-22		Br	II (≥1600)	400
FIP-23		Pl, Li, Ki	II (≥1600)	≥1600
FIP-24		As, CSF, Ki	I (≥1600)/II (≥1600)	800
FIP-25			I (≥1600)	10
FIP-26			I (400)	≤10
FIP-27			I (400)	10
FIP-28			I (800)	≤10
FIP-29			I (200)/II (400)	≥1600
FIP-30			I (800)/II (≥1600)	≥1600
FIP-31			I (400)	≤10
FIP-32			I (400)	≤10
FIP-33			I (800)	≤10
FIP-34			I (≥1600)	10
FIP-35			I (800)	≤10
FIP-36			I (400)	≤10

^a^: *R*, Rectal swabs; *As*, Ascites; *Pl*, Pleural effusions; *B*, Blood; *CSF*, Cerebrospinal fluid; *Li*, Liver; *Lu*, Lung; *Ki*, Kidney; *Br*, Brain.

^b^: NT titer against a type II FIPV.

### Association between FCoV genotype and serotype

Among seropositive cats, 70.4% (202/287) of cats had antibodies specific to rRBD-I, whereas 3.5% (10/287) had antibodies to rRBD-II, and 17.8% (51/287) reacted to both types (Table 1). Infection with serotype I FCoV appeared to be dominant in both the FIP and the non-FIP groups (Table 1).

To evaluate the correlation between the serotype and the genotype of the infection, plasma samples collected from type I or II FCoV-infected cats with FIP were analyzed. In comparison of results derived from serotyping and genotyping, the serotype of FCoV was found highly agree with the genotype in most cases. For the cats with type I FIP, most (17/20, 85%) had antibodies specific to rRBD-I, except for three cats (FIP-18, FIP-19 and FIP-20) (Table 2). Of the solely type II-infected cats with FIP, four cats (FIP-21 to FIP-24) had titers specifically against rRBD-II, but only one of these cats (FIP-24) possessed antibodies against rRBD-I as well (Table 2).

Of the cats without FIP with type I FCoV detected by RT-nPCR, 78.1% (25/32) had antibodies against r-RBD-I, and 9.4% (3/32) were positive for antibodies against both types. Additionally, the serotypes of four samples failed to be identified. Moreover, three plasma samples collected from healthy cats with type II FCoV detected in fecal samples were used for serotyping [16]. All three samples were FCoV seropositive, but only one was identified as infected by both types of FCoV. Of those cats infected with the FCoVs that failed to be genotyped, 84.6% (22/26) possessed antibodies against type I FCoV (Table 3).

**Table 3:** Correlation between serotype and genotype in FCoV antibody-positive animals without FIP

**RT-nPCR**	**Genotype**	**Serotype n (%)**				**Total**
		**I**	**II**	**I/II**	**Negative**	
Positive	I	25 (78.1)	0 (0)	3 (9.4)	4 (12.5)	32
	II	0 (0)	0 (0)	1 (33.3)	2 (66.7)	3
	UT	22 (84.6)	1 (3.8)	2 (7.7)	1 (3.8)	26
	NA	23 (56.1)	1 (2.4)	13 (31.7)	4 (9.7)	41
Negative		24 (75)	1 (3.1)	3 (9.4)	4 (12.5)	32
NA		55 (68.8)	2 (2.5)	15 (18.7)	8 (10)	80
Total		149 (69.7)	5 (2.3)	37 (17.3)	23 (10.7)	214

*UT*: Untypable.

*NA*: Not available.

## Discussion

In this study, an FCoV type-specific, recombinant protein-based IFA was established. The expressed recombinant RBD region of the type II FCoV S protein, bearing one-tenth of the authentic S protein critical in both virus neutralization and cell attachment, is highly conserved among FCoV, TGEV and CCoV [17]. The alignment of the amino acid sequences of this region revealed relative conservation among prototypic viral strains within the same serotype (i.e., 86.8-95.4% and 91.4-98.3% of type I and II FCoV, respectively) but were distinct between different serotypes (20.7-23.0%) (Figure 1a). As the expressed partial S proteins contained four N-linked glycosylation sites for both type I (551-NVT-553, 559-NDT-561, 591-NGS-593 and 596-NVT-598) and type II (520-NKS-522, 536-NIT-539, 558-NIT-560 and 566-NNT-568) FCoV (Figure 1a), the baculovirus expression system was chosen to provide post-translational modification and complex folding, including glycosylation [18],[19]. A similar strategy was applied to severe acute respiratory syndrome (SARS)-CoV, and the recombinant RBD maintained authentic antigenicity that induced a strong RBD-specific antibody response [20]. In addition, baculovirus-expressed, truncated SARS-CoV S protein-based IFAs were found to be highly sensitive and specific compared with conventional whole virus-based IFAs [21],[22].

Based on different testing methods, the prevalence of FCoV in field cats varies among countries, e.g., 50% in Switzerland [10], 34% in Australia [23], 22% in Japan [11] and 13.7% in Korea [24]. In the present study, FCoV-specific antibodies detected by IFA based on proteins derived from a local FCoV isolate revealed a seroprevalence of 34.5%. Previous studies revealed that >80% of seropositive cats are suspected to be infected with type I FCoV [9]-[11]. In the present study, 70.4% of local cats were infected with type I FCoV. Infection with type II FCoV in Taiwan (21.3%, 61/287) is higher than in other countries, i.e., 4.4% in Switzerland and 10.1% and 2% in Japan in 1992 and 2007, respectively. The dense population and close contact between cats and other animals in Taiwan could be reasons contributing to the higher rate of recombination between FCoVs and CCoVs or other alphacoronaviruses, leading to a relatively high type II FCoV infection rate. This relatively high antibody titer against type II virus might also result from other alphacoronavirus infection, as feline cells are susceptible to infection with FCoV, CCoV, TGEV and HCoV-229E [25] and given that seroconversion was observed in cats experimentally infected with CCoV [26], TGEV [27] or HCoV-229E [28].

In a prior study, a competitive ELISA was used to discriminate the serotype of infection [11]. However, certain sera could not be typed once inhibition reached 30-80%. In the present study, serotypes were determined by the direct observation of a fluorescent signal specifically indicating two types of recombinant protein, which decreased the ambiguity. In another IFA study using type I or type II FCoV-infected cells, 23% of cats displayed equal titers against type I and II FCoV. The phenomenon was suggested by the authors to result either from antibodies against common antigenic epitopes in other structural protein or from co-infection with the two viruses [10]. Our type-specific S protein-based IFA can avoid the sharing of common antigenicity, and co-infection can be readily identified.

This is the first report to correlate viral infection with the specific antibody evoked. We compared the serotypes and the genotypes of FCoV-infected animals. Most type I FCoV-infected cats with FIP harbored anti-type I FCoV antibodies, and three of them displayed antibodies against both types of FCoV, indicating co-infection with FCoV and/or other CoVs. Additionally, one cat with type II FIP (FIP-24) displayed an antibody response to type I and II FCoV simultaneously. This finding matches the recombination theory that states that type II FCoV arises from the mutation of type I FCoV. However, we found that three cats with type II FIP possessed antibodies solely against type II FCoV. Among those cats, FIP-23 (cat 11) was living in a shelter that had experienced a recent FIP outbreak caused by type II FCoV [29]. The presence of antibodies solely against type II FCoV in cat FIP-23 provides an additional clue about the horizontal transmission of type II FIP. In the case of cat FIP-22, sequencing analysis of the C-terminal one-third of the viral genome indicated a virus bearing high resemblance to CCoV, but not to FCoV. Combining the serological and genetic findings, this FIP could have resulted from a CCoV infection. Further genetic analysis regarding this speculation is currently under investigation.

Among healthy cats with untypable FCoV, 84.6% were found to have a type I FCoV infection. Although the serotype of the antibodies detected did not always match the virus present at the time of sampling, infection with FCoVs, and especially with type I, is persistent and can last for several years in a multicat environment [14],[30]. Moreover, type I virus has been reported to be more genetically diverse than type II virus in the genotyped region, and this diversity might hinder PCR amplification [16]. Despite the better distinguishability, 8.3% of the cats in the current study possessed anti-FCoV antibodies that could not be serotyped. This issue could have been due to the lower antibody titers against FCoV, or polymorphisms may exist in the target region in S protein.

FCoV antibody titers when accurately performed are thought to be of some value in distinguishing enteric infection from FIP. Cats with IFA titers ≥1:3200 are highly suggestive of FIP [31]. In our study using the serotyping method established, type-specific FCoV antibody could be titrated and the titer in some of our FIP cats reached relatively high level as well (≥1:1600). Our recent study revealed that the infection of serotype II FCoV correlated significantly with the occurrence of FIP [16]. Also horizontal transmission of a serotype II virus was clearly demonstrated to responsible for a FIP outbreak [29]. Continuous survey of the antibodies against two serotype of FCoV, especially in the multi-cat environment could help in identifying the invasion of any type II FCoV hence isolating the animals to prevent future FIP occurrence.

## Conclusions

In conclusion, compared with the results of previous studies, we present a relatively simple serotyping method to discriminate between two types of FCoV infection. Based on this method, type I FCoV was found to be predominant compared with type II in Taiwan. This survey also provided clues supporting the evolution and horizontal transmission of type II FIPV. The epidemiological survey also provided valuable information for disease control and vaccine development.

## Competing interests

The authors declare that they have no competing interests.

## Authors’ contributions

YTW performed the sampling and preparation, FCoV detection, genotyping, type-specific IFA development, anti-FCoV antibody detection, serotyping and further analysis and prepared the manuscript. LLC conceived the study, participated in study design and coordination and contributed to the preparation of the manuscript. CHW participated in study design, supervised the type-specific IFA development and contributed to the preparation of the manuscript. All authors read and approved the final manuscript.
